# Thinking caps for everyone? The role of neuro-enhancement by non-invasive brain stimulation in neuroscience and beyond

**DOI:** 10.3389/fnsys.2014.00071

**Published:** 2014-04-29

**Authors:** Felix Duecker, Tom A. de Graaf, Alexander T. Sack

**Affiliations:** ^1^Department of Cognitive Neuroscience, Faculty of Psychology and Neuroscience, Maastricht UniversityMaastricht, Netherlands; ^2^Maastricht Brain Imaging Center, Maastricht UniversityMaastricht, Netherlands

**Keywords:** neuro-enhancement, mechanisms of enhancement, non-invasive brain stimulation, transcranial magnetic stimulation, transcranial current stimulation

## Abstract

Neuro-enhancement by non-invasive brain stimulation (NIBS) has recently made considerable progress, triggering discussions regarding future applications to enhance human performance. We show that neuroscientific research does not aim at improving brain functions *per se*. Instead, neuro-enhancement is a research tool that has great potential to reveal the neural mechanisms underlying perception, cognition, and behavior. We provide instructive examples that showcase the relevance of neuro-enhancement by NIBS in neuroscience. Importantly, we argue that the scientific value of neuro-enhancement critically depends on our understanding of why enhancing effects occur. This is in contrast to applications of neuro-enhancement in other domains, where such knowledge may not be required. We conclude that neuro-enhancement as a therapeutic tool or in healthy people outside of neuroscience should be kept conceptually distinct, as these are separate domains with entirely different motives for enhancing human performance. Consequently, the underlying principles that justify the application of NIBS will be different in each domain and arguments for or against neuro-enhancement in one domain do not necessarily generalize to other domains.

The suggestion to “put on your thinking cap” is generally seen as purely metaphorical. Recent advances of non-invasive brain stimulation (NIBS) techniques have created the prospect of real “thinking caps” that might have the potential to improve perception, cognition, and behavior. Outside the scientific community, these developments have been often interpreted as reflecting the ambition to enhance human performance *per se*, leading to heated debates on applicability, desirability and morality of neuro-enhancement. And indeed, people should carefully consider whether, how, and when the application of NIBS to enhance brain function is appropriate (Farah et al., 2004; Cohen Kadosh et al., 2012). We here argue that in neuroscientific research, the enhancement of brain function serves as a means to an end, that is, it aims at gaining insights into brain function. The scientific value of neuro-enhancement therefore critically depends not on the fact *that* enhancing effects occur, but on our understanding of *why* they occur. This is in contrast to applications of neuro-enhancement in other (non-academic) domains where such knowledge may be not required. In this article, we first explain the relevance of neuro-enhancement by NIBS for neuroscience and showcase how it has produced valuable insights into how the brain works. Then, we outline the different domains that neuro-enhancement by NIBS could be considered for. Rather than making any judgments on the moral or ethical justifications of neuro-enhancement here, we argue that the debate on such matters should be held separately for each of these domains. From this follows that arguments against neuro-enhancement in one domain do not necessarily apply to other domains.

## Effects of NIBS

The growing popularity of NIBS is due to the fact that induced brain changes have been shown to be perceptually, cognitively, and behaviorally relevant. In other words, NIBS can affect everything from low-level vision (Amassian et al., 1989) to attention (Duecker et al., 2013) to social-economic behavior (Knoch et al., 2006). The most common NIBS techniques are transcranial magnetic stimulation (TMS), transcranial current stimulation (tCS) with either direct (tDCS) or alternating (tACS) currents, and transcranial static magnetic stimulation (tSMS). TMS involves the administration of magnetic pulses to localized brain areas. The effects of single pulse TMS are short-lasting and can affect ongoing neuronal processes whereas rhythmic pulse sequences can yield long-lasting effects on the human brain (see e.g., Hallett, 2007 for a TMS primer). In contrast, tCS is applied over larger areas of cortex to send an electrical current through brain matter (see e.g., Paulus, 2011a for a tCS primer). Finally, tSMS exposes the brain to a static magnetic field by positioning a magnet on the head (Oliviero et al., 2011; Paulus, 2011b). Simply speaking, these techniques produce a combination of excitatory and inhibitory effects at the neuronal level. The polarization of neurons is changed and, depending on the stimulation parameters, regional cortical excitability either increases or decreases. Many different stimulation protocols have been developed over the years and it is common practice to label protocols as either inhibitory or excitatory. It is very tempting to directly relate these effects on cortical excitability to changes in brain function such that excitatory protocols necessarily lead to neuro-enhancement. However, this would be an oversimplification. Whether a particular NIBS protocol will have enhancing or impairing effects on the perceptual, cognitive, or behavioral level will depend not only on excitability changes but also on the functional properties and underlying mechanisms of all brain areas involved, as well as the interactions between them. This is exactly why both neuro-disruption and neuro-enhancement effects are scientifically valuable; because in the proper theoretical framework they allow us to begin teasing apart this functional neuronal architecture. Due to this complexity, we will here not provide an overview of all available NIBS protocols but will instead present mechanisms of neuro-enhancement with instructive examples mostly drawn from the attention literature.

## Accidental neuro-enhancement

Interestingly, NIBS was initially rarely conceptualized as a neuro-enhancing method but was instead applied with the aim to disrupt brain activity. The idea was to reveal a causal structure-function relationship through a NIBS-induced behavioral deficit, indicating the functional necessity of the stimulated brain area for normal task performance (Sack, 2006). Enhancing effects were very uncommon and their occurrence was rather incidental than purposefully induced. For example, Walsh et al. (1998) showed that TMS over hMT+/V5 lead to perceptual enhancements as reflected by improved performance on a visual search task when motion was either absent or task-irrelevant. Similarly, improvements in cognitive performance have been found in the context of picture naming (Mottaghy et al., 1999) and other language-related processes (Epstein, 1998). As most authors acknowledged, interpretation of these results was difficult because a theoretical framework to explain such findings was often lacking.

## Purposeful neuro-enhancement

Once the potential to enhance brain function by NIBS was recognized, the effects of many different NIBS protocols were explored and, unsurprisingly, excitatory protocols often led to improved performance (Coffman et al., 2014; Luber and Lisanby, 2014). This research line has clearly shown that NIBS is capable of improving various brain functions including perception, attention, memory, and even acquisition of skills that are highly relevant for everyday life such as numerical abilities (Cohen Kadosh et al., 2013) and arithmetic (Snowball et al., 2013). In some sense, this is very similar to the original approach of revealing causal structure-function relationships outlined above. After all, also enhancing effects do imply a functional role of the stimulated brain area in a particular process. However, when the underlying mechanisms that cause an enhancement remain unknown, enhancement results, as exciting as they may be, can be of limited scientific value when not followed-up by further investigations. As will be argued below, NIBS primarily delivers its full potential when embedded in scientific theory. It then produces strong direct evidence regarding the mechanisms underlying brain functions.

## Inter-hemispheric competition revealed with NIBS

The application of NIBS has been particularly successful in the context of attention research. Neuroimaging studies had already produced detailed knowledge regarding the brain networks involved in attentional control (Corbetta and Shulman, 2002) but struggled to differentiate between several competing models that were based on lesion studies in neglect patients (Kinsbourne, 1977; Heilman and Abell, 1980; Mesulam, 1981). Because these models were originally developed to explain attentional deficits after brain damage, it was relatively easy to derive hypotheses regarding the consequences of NIBS on attention in healthy people. Interestingly, one of these models also predicted enhancing effects of NIBS, namely Kinsbourne’s (1977) “opponent processor” model. It stated that each hemisphere has a natural attention bias to the contralateral side of space. Under normal conditions, the two hemispheres are kept in balance due to inter-hemispheric competition. Whenever an imbalance between the hemispheres occurs, attention will be biased towards one side of space. In the context of neuro-enhancement, there are two important aspects to this model. First, in a situation of inter-hemispheric competition, behavioral enhancement and impairment are predicted to be two sides of the same coin. When the overall attention bias is directed towards one hemifield, processing for stimuli on that side of space will be improved at the expense of impaired processing for stimuli on the other side of space. Specifically, any change in excitability of one hemisphere will always affect its inhibitory influence on the other hemisphere as well, so that the final imbalance between the hemispheres is determined by the interaction between them. Second, such an imbalance can be induced in different ways, either by facilitating or inhibiting one hemisphere. As already explained above, this local excitability change then also affects the other hemisphere where the opposite effect occurs. Thus, this model lends itself perfectly for being tested with NIBS. Hilgetag et al. (2001) were among the first to directly test Kinsbourne’s “opponent processor” model. They applied 1 Hz repetitive TMS over right or left parietal cortex and assessed performance on a target detection task. As one might expect with a protocol that decreases cortical excitability, target detection was impaired in the contralateral hemifield. In addition, however, they also observed enhanced target detection in the ipsilateral hemifield, strongly supporting the notion of inter-hemispheric competition. Importantly, these results make perfect sense in the light of Kinsbourne’s “opponent processor” model but would be puzzling and less informative if this model did not exist. In other words, in and of itself the enhancement of ipsilateral processing was an interesting trivia. But in the appropriate theoretical framework, the enhancement result became neuro-scientifically valuable. Similarly, and corroborating this finding, Dambeck et al. (2006) found a contralateral impairment of target detection with single-pulse TMS over right or left parietal cortex. Strikingly, when applying TMS over both hemispheres simultaneously, the behavioral effects of unilateral TMS disappeared and performance was back to normal because the balance between hemispheres remained unchanged. Again, this seemingly paradoxical result is turned into meaningful insights into the mechanisms underlying attention control when linked to the appropriate theoretical framework. As pointed out before, the effects of NIBS are not simply determined by the stimulation protocol but are also a consequence of the functional architecture of the brain. In this sense, these findings are extremely valuable as they can be directly related to competing models of attentional control.

## Entrainment and phase-coupling

Thus far, NIBS-induced changes of cortical excitability have been conceptualized as rather static effects. However, it is well-established that rhythmic patterns of neural activity are an essential aspect of information processing in the brain. In the context of attention, the power and phase of alpha-band activity in occipito-parietal regions has repeatedly been related to attentional/perceptual performance (Jensen and Mazaheri, 2010; Klimesch, 2012). Alpha power is negatively correlated with perceptual performance (van Dijk et al., 2008) and lateralized when shifting attention to one hemifield (Händel et al., 2011). It is possible to “entrain” alpha-band activity in the brain using rhythmic sensory stimulation (e.g., de Graaf et al., 2013), but also directly and locally, using non-invasive brain stimulation (e.g., Romei et al., 2010; Thut et al., 2011), in order to investigate frequency-dependent modulations of task performance. Romei et al. (2010) applied a short burst of rhythmic TMS at alpha frequency, or flanker frequencies (theta or beta), prior to stimulus presentation. Only for alpha-frequency stimulation, target visibility in the contralateral visual field was reduced, while it was enhanced in the ipsilateral hemifield. So as above, impairment and enhancement co-occurred due to inter-hemispheric competition, but this time in a frequency-dependent way. Recent studies have pushed even further, demonstrating that not only the frequency of oscillations but also their phase can be essential for neural processing. Polanía et al. (2012) used tACS with electrode patches on frontal and parietal cortex both connected to a third reference electrode on the vertex. Previously acquired EEG results revealed a 0-degree phase lag in synchronized activity in the theta band between frontal and parietal cortex during a working memory task. When frontal and parietal regions were stimulated with an oscillating current pattern at a similar frequency, the phase lag between the frontal and parietal stimulation determined working memory performance. This highly advanced NIBS protocol yielded a fairly simple finding: stimulation of the fronto-parietal network in sync enhanced working memory performance whereas out of sync stimulation impaired working memory performance. This has deep and intriguing implications for our understanding of brain function and, together with the other examples described above, demonstrates how far NIBS has come as a research tool. It can produce neuro-enhancing effects, but its scientific power lies in revealing the neural mechanisms underlying perception, cognition, and behavior. And the growing complexity of NIBS approaches enables increasingly meaningful results, increasingly strong conclusions, and increasingly specific hypotheses about functional brain architecture.

## Neuro-enhancement beyond neuroscience

The overview we presented in this article has focused on neuro-enhancement as a research tool, mainly taking brain mechanisms underlying attention as an example, and illustrated how it can produce valuable insights into the neural mechanisms underlying human behavior and cognition. We outlined the various forms neuro-enhancement can take, the various experimental settings underlying them, and the many valuable neuroscientific insights one could gleam from it. Importantly, in the neuroscientific domain neuro-enhancement by NIBS mainly serves its purpose when embedded in theoretical models of the brain. Enhancing effects that lack any explanation are of very limited scientific value and require further attempts to unravel the underlying mechanisms.

Beyond neuroscience, however, the application of NIBS for neuro-enhancement is not necessarily motivated by its scientific value. Instead, enhancing perception, cognition, and behavior could, for some, be considered a goal in itself *irrespective of the underlying mechanisms* that produce such effects. That would be neuro-enhancement as an endgoal, rather than as a means to an end. We therefore suggest that the current debate concerning application of neuro-enhancement should be distinguished for different domains. Specifically, we propose three domains that should be kept separate, at least to some extent, namely neuro-enhancement (a) as a research tool; (b) as a therapeutic tool and (c) applied in healthy people outside of neuroscience.

Note that we certainly do not argue for a “hands-off” approach of scientists to the larger debate on desirability of neuro-enhancement in general. After all, it is undeniable that clinical or non-academic applications of neuro-enhancement stem directly from the efforts in the academic domain. If the science doesn’t first develop the tool, there is no tool to be applied outside the scientific setting. What we argue for instead is to have the debate in all domains, but to keep in mind clearly which domain we are discussing. We should keep an open mind to the possibility that the ethical, moral, and practical conclusions that may flow from the larger neuro-enhancement debate will be different for each of the three domains. At present, some neuro-enhancing approaches of NIBS have the potential to be applied as a therapeutic tool in patients, and results so far are promising (Hummel and Cohen, 2006; Miniussi et al., 2008). Still, most neuro-enhancing effects of NIBS appear to be of very limited practical relevance in everyday life. But as the field progresses the possible applications of NIBS will increase. People may have serious concerns about such possible future application of NIBS to healthy human brains in schools, universities, or the workplace. A debate would ensue whether or not society should desire, or even allow, such practices. Should companies be allowed to have their employees wear “thinking caps” to boost performance? Would they even be allowed to demand it from their workers? These are relevant questions that should be discussed by laypeople, government, and scientists. They are extreme examples in a sense, but they allow us to highlight the key point here, which is that the different domains where neuro-enhancement is now or in the future applicable should be considered separately in discussions about neuro-enhancement, its value, its risks, its desirability, its development and its general pursuit. Neuroscientists should participate in this discussion, contributing their expertise. But laypeople should participate as well, since law- and policy-makers need to develop rules and regulations on the basis of both expert opinion and societal support. But such rules and regulations should, in our view, be specific to the different domains. If the debate takes shape according to these lines, we believe that neuro-enhancement can continue to be of great value for our understanding of the brain, of potential use in clinical and therapeutic environments, and perhaps in the future applied responsibly in non-academic settings.

## Conflict of interest statement

The authors declare that the research was conducted in the absence of any commercial or financial relationships that could be construed as a potential conflict of interest.
